# Chilblain lupus induced by infliximab therapy

**DOI:** 10.1093/rap/rkaf027

**Published:** 2024-03-05

**Authors:** Priyanka Lakhani, Huw Beynon, Richard Stratton

**Affiliations:** Department of Rheumatology, Royal Free Hospital, London, UK; Department of Rheumatology, Royal Free Hospital, London, UK; Department of Rheumatology, Royal Free Hospital, London, UK

A 74-year-old woman with ulcerative colitis was started on infliximab. Six months later, after the infliximab dose was increased, she gradually developed a rash, arthralgia, myalgia and fatigue. Her ulcerative colitis was in remission and she had no previous rheumatological history. Examination revealed red and dusky purple patches and plaques across the fingers and palms, consistent with chilblains (Fig. 1). Furthermore, a symmetrical polyarthritis with synovitis of the small digits of the hands and wrists was evident. CRP was elevated at 30 mg/dl and the ESR was 26 mm/h. An ANA test was positive, with a 1:320 homogeneous pattern (no previous ANA result). aCL antibody was positive and anti-dsDNA and extractable nuclear antigen were negative. Full blood count, complement levels and liver and renal profiles were normal. Urinalysis was bland. A diagnosis of TNF-α inhibitor–induced chilblain lupus was made. Treatment included cessation of infliximab and intravenous methylprednisolone, with a major improvement in symptoms. Rarely, drug-induced lupus presents as chronic cutaneous lupus erythematosus, including chilblain lupus. To date, there are only 11 published cases of chilblain lupus as a presentation of anti-TNF-α-induced lupus [1]. This case highlights the rarer adverse effects of anti-TNF-α agents that must be considered in a patient presenting with chilblain lesions.

**Figure 1.**
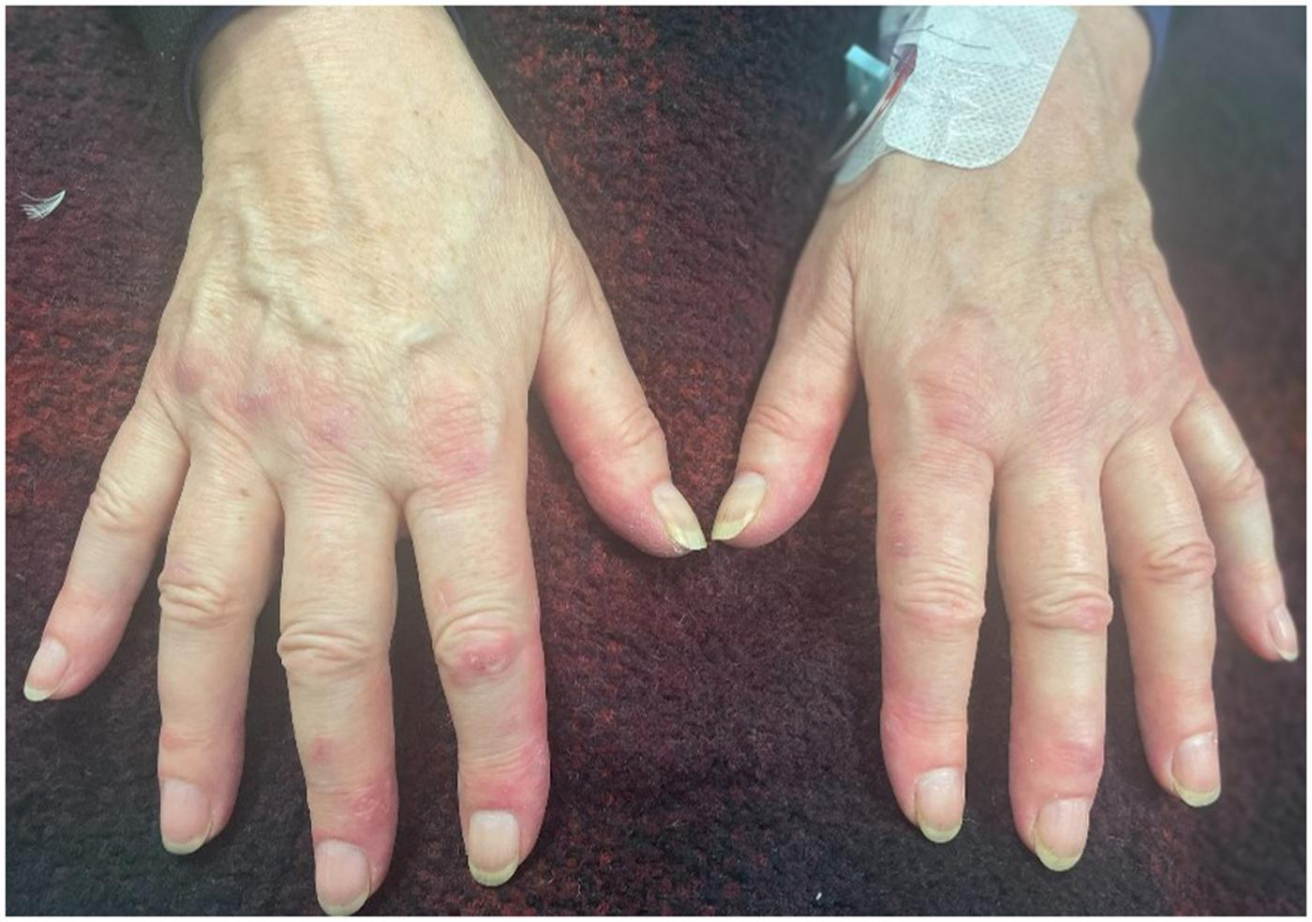
Chilblain lesions and small joint polyarthritis in a patient treated with a TNF-α inhibitor

## Data Availability

No new data were generated or analysed in support of this research.
